# Effect of Tai Chi-Based Psychosomatic Rehabilitation Exercise on Physiological Function and Mental Health of Patients with Coronary Heart Disease: A Meta-Analysis

**DOI:** 10.31083/j.rcm2506227

**Published:** 2024-06-24

**Authors:** Chu Fan, Hangyu Yan, Kehang Lei, Xuepeng Li, Dan Li, Shutong Dong, Yue Zhang, Yutong Cheng, Zhao Li, Nan Li, Zhizhong Li, Ji Huang

**Affiliations:** ^1^Department of Cardiology, Beijing AnZhen Hospital, Capital Medical University, National Clinical Research Center for Cardiovascular Diseases, Office of Beijing Cardiovascular Diseases Prevention, 100000 Beijing, China

**Keywords:** coronary heart disease, rehabilitation, Tai Chi, meta-analysis

## Abstract

**Background:**

Tai Chi is an increasingly utilized aerobic rehabilitation exercise in the field of cardiovascular disease (CVD). However, there remains debate regarding its effects on physiological function and mental health in patients with coronary heart disease (CHD). This study aims to investigate the impact of Tai Chi-based rehabilitation exercises on physical and psychological health outcomes for CHD patients.

**Methods:**

By collecting data from 12 databases up to December 2022, we conducted a meta-analysis of randomized controlled trials (RCTs) to evaluate the effects of Tai Chi on the physical function and psychological health among CHD patients.

**Results:**

We analyzed twenty qualified studies involving 2095 patients. Meta-analyses revealed that compared with conventional therapy groups, those who participated in Tai Chi-based interventions demonstrated significant improvements in physical function as measured by six-minute walk test (6MWT) [mean difference (MD) = 56.40, 95% confidence interval (CI) (38.50, 74.29), *p* < 0.01], maximal oxygen consumption (${\mathrm{VO}}_{2}$ max) [standardized mean difference (SMD) = 0. 57, 95% CI (0.12, 1.03), *p* = 0.01], New York Heart Association (NYHA) class [relative risk (RR) = 1.34, 95% CI (1.18, 1.53), *p* < 0.01] and physical health components (PHC) [SMD = 1.23, 95% CI (0.76, 1.69), *p* < 0.01]. Additionally, Tai Chi participants showed greater improvement than control groups across various psychological parameters including anxiety scales [SMD = –0.80, 95% CI (–1.33, –0.28), *p* = 0.003], depression scales [SMD = –0.77, 95% CI (–1.32, –0.23), *p* = 0.005] and mental health components (MHC) [SMD = 1.27, 95% CI (0.76, –1.78), *p* < 0.01]. The GRADEpro (Grade Guideline Development Tool) indicated evidence levels ranging from very low to moderate.

**Conclusions:**

The present meta-analysis demonstrates that mind-body rehabilitation exercises based on Tai Chi can improve both physical and psychological health outcomes for CHD patients. These findings suggest that this exercise pattern may be a potential option for cardiovascular rehabilitation.

**PROSPERO Registration:**

The protocol for this systematic review and meta-analysis has been registered with PROSPERO International Prospective Systematic Reviews (No: CRD42022370021, http://www.crd.york.ac.uk/PROSPERO).

## 1. Introduction

Since the onset of the 21st century, there has been a substantial surge in the prevalence of cardiovascular diseases (CVD) [1]. According to the data from World Health Organization, CVD account for over 17.3 million death annually, representing approximately 30% of global mortality rates. Among these fatalities, coronary heart disease (CHD) alone is responsible for around 7.4 million deaths [2]. Extensive research has demonstrated a strong correlation between physiological function and mental well-being concerning the developing and progressing of CHD [3, 4]. The deterioration of physiological function and presence of emotional disorders serve as powerful predictors for poor prognosis in patients with CHD. Consequently, it is becoming increasingly important to consider both physical and psychological diagnoses and treatments when dealing with individuals with CHD. Effective cardiac rehabilitation exercise can not only enhance patients’ exercise tolerance, cardiac function and overall quality of life but also reduce the incidence of major adverse cardiovascular events (MACEs), while alleviating psychological issues such as anxiety and depression [5]. However, identifying which specific cardiac rehabilitation exercises are truly effective remains an ongoing inquiry that requires constant exploration of the optimal approaches [6]. Tai Chi is an emerging exercise modality within rehabilitation programs that seamlessly integrates physical exercise with mental relaxation-thus encompassing both physical and psychological treatment aspects simultaneously. Originating from traditional Chinese medicine principles including meridians, acupoints, Qi and blood circulation guidance techniques, and visceral manifestation [7], Tai Chi is now being explored as a potential option for cardiac rehabilitation in select regions [8]. However, there are still debates in clinical practice regarding whether an integrated approach to physical and mental rehabilitation can improve the prognosis of patients with CHD or be widely promoted as a mode of cardiac rehabilitation. The aim of this article is to conduct a meta-analysis and systematic evaluation of randomized controlled trials (RCTs) investigating the effects of Tai Chi exercise intervention on patients with CHD.

## 2. Methods

The protocol for this systematic review and meta-analysis has been registered with PROSPERO International Prospective Systematic Reviews (No: CRD42022370021, http://www.crd.york.ac.uk/PROSPERO).

### 2.1 Availability of Data, Materials and Search Strategies

In order to comprehensively identify all relevant RCT studies published since the establishment of each database until December 25th 2022, two primary authors independently conducted systematic searches across 12 electronic databases, including PubMed, Scopus, OVID, Cochrane Library, Web of Science, Embase, ProQuest, CNKI, CBM, DuXiu, VIP, WanFang. Additionally, a manual search was performed on the retrieved articles to obtain additional references. If any literature content was incomplete or reports were unavailable, the relevant authors were contacted via email for further information. No restrictions were applied regarding publication year or language. A combination of subject headings and free words was utilized in the search strategy encompassing terms such as coronary heart disease; coronary artery disease; coronary atherosclerosis; percutaneous coronary intervention; coronary artery bypass surgery; myocardial infarction; acute coronary syndrome; Tai Chi, Tai Chi Chuan and randomized controlled trial. An illustrative example of the search conducted in PubMed can be found in **Supplementary Material 1**.

### 2.2 Inclusion and Exclusion Criteria

Inclusion criteria of this study was based on the following factors: study design, population characteristics, interventions and outcome indicators. (1) The study design specifically focused on randomized controlled trials without any restrictions on allocation concealment or blinding; (2) The study population consisted of patients diagnosed with CHD, regardless of disease severity or stage; (3) The experimental group received Tai Chi rehabilitation exercises in addition to routine treatment. There were no limitations regarding the type, duration, frequency, or intensity of the Tai Chi exercises. The control group may either routine treatment without any exercise intervention or other non-Tai Chi rehabilitation types such as aerobic or resistance exercises (e.g., walking, biking, aerobics, elastic band resistance exercise); (4) Outcome measures were categorized into physiological and mental health domains. Physiological outcome measures included six-minute walk test (6MWT), maximal oxygen consumption (${\mathrm{VO}}_{2}$ max), New York Heart Association (NYHA) class and physical health components (PHC) scores; while mental health outcome measures encompassed anxiety scale scores, depression scale scores and mental health components (MHC) scores. Exclusion criteria comprised: literatures with repeated publication or multiple publications; non-randomized controlled trials or mismatched experimental designs; literature where the study population did not meet the established inclusion criteria; literature with incomplete data or significant baseline difference between the control group and the study; literature consisting of systematic reviews along with experimental plans and quantitative analyses.

### 2.3 Data Extraction

The Cochrane Handbook for Systematic Reviews of Interventions was utilized to conduct a comprehensive literature screening and data extraction process [9]. Subsequently, the literature was imported into Endnote software (version 9, Thomson Corporation, Stanford, CT, USA), where two researchers independently and in a double-blind manner screened it based on predetermined criteria. Any discrepancies in data extraction were resolved through discussion or referee to a third party for adjudication. The extracted data underwent rigorous cross-checking procedures. The data extraction encompassed various aspects including fundamental information (e.g., first author’s name, publication date, study site, study ID and databases), methodological quality assessment (risk of bias scale), characteristics of the study subjects (inclusion criteria, exclusion criteria, sample size, age distribution, gender ratio), details regarding the intervention (subgroup status, type, duration/frequency/period), control measures employed in the studies conducted as well as outcomes reported (dropout rates and handling of missing data). Additionally, outcome indicators (6MWT, ${\mathrm{VO}}_{2}$ max, NYHA class, PHC score, anxiety scale, depression scale and MHC score) were also included.

### 2.4 Outcome Definition

6MWT measures the maximum distance a patient can walk at their tolerated speed on a flat, rigid surface within a span of 6 minutes [10]; ${\mathrm{VO}}_{2}$ max represents the amount of oxygen consumed by the human body after engaging in exercise at maximum intensity, indicating when the body exhibits weakness and is unable to sustain further physical activity [11].

The NYHA classifies cardiac function impairment based on heart failure symptoms [12], utilizing a four-stage classification system. Improvement in NYHA class is categorized as effective or ineffective. Effective improvement signifies controlled heart failure or an improvement of at least one grade in cardiac function. Ineffective improvement refers to less than one grade improvement or deterioration in cardiac function. PHC encompasses four dimensions: physical health (PF) score, role physical (RP) score, bodily pain (BP) score and general health (GH) score [13]. The anxiety scale is a standardized tool used for assessing anxiety levers during psychological evaluations, quantifying symptom intensity and monitoring fluctuations throughout treatment [14]. Similarly, the depression scaleserves as a standard tool for evaluating depressive states, measuring severity and changes during treatment. The MHC score comprises four dimensions of mental health-related SF-36 scores: vitality (VT), social function (SF), emotional function (RE), and mental health (MH) [13].

### 2.5 Quality Assessment

The risk of bias (ROB) tool from RevMan (version 5.3, Cochrane Groups, Copenhagen, Denmark) was utilized to assess the risk of bias in the included literature, following the criteria for ROB assessment outlined in the Cochrane Handbook [9]. The study quality was evaluated across seven dimensions: random sequence generation (selection bias), allocation concealment (selection bias), blinding of participants and personnel (performance bias), blinding of outcomes assessment (detection bias), incomplete outcome data (attrition bias), selective reporting (reporting bias), and other bias. Quality assessments during the statistical analysis were categorized as follows: studies with five or more items were considered low risk of bias, three to four items considered moderate risk of bias, and less than three items considered high risk of bias. Two investigators independently conducted the quality assessment, namely C-F & HY-Y. In case of disagreement, a third party named D-L was consulted for resolution.

### 2.6 Statistical Analysis

The literature was analyzed using RevMan 5.3 software provided by the Cochrane Collaboration. Continuous variable outcomes were expressed as mean difference (MD) if they were based on the same measurement method or unit, and as standardized mean difference (SMD) if they were measured by different methods or units. Following Cohen’s conventional interpretation (1988), SMD values of ≤0.2, 0.20 < SMD ≤ 0.80 and ≥0.8 were considered small, moderate, and large effects respectively. Outcomes of categorical variables were expressed as relative risk (RR). MD, SMD and RR with 95% confidence intervals (CIs) are reported. All reported *p* values are two-sided and *p* < 0.05 was considered to indicate statistical significance. Heterogeneity among studies was assessed using $I^{2}$ test. If $I^{2}$ ≤ 50% and *p* > 0.1, it indicated good homogeneity and the fixed effect model was employed; Conversely, if $I^{2}$ > 50% and *p* ≤ 0.1, significant heterogeneity was observed necessitating the use of a random effect model [15]. Due to the limited study numbers in each reference included, no funnel plot analysis was conducted to detect publication bias. To minimize bias in our evaluation of included studies including risk of bias assessment, inconsistency (heterogeneity), indirectness, imprecision, and publication bias we utilized GRADEpro online software (version 3.6, Cochrane Groups, Cavendish, London, UK) [16]. The level of evidence for each study was classified as very low, low medium or high.

## 3. Results

### 3.1 Literature Screening

**Fig. 1.**
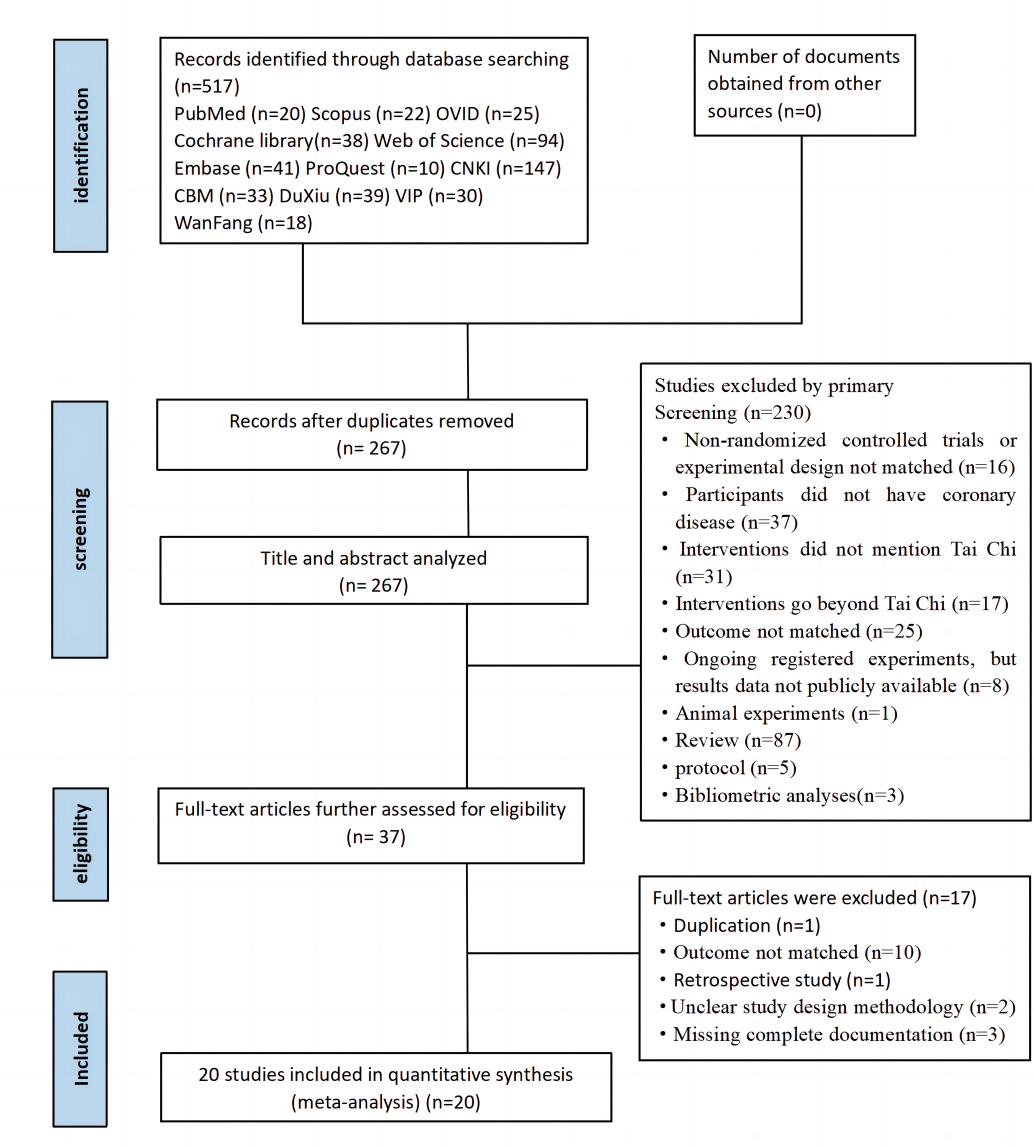
**Literature screening process**.

The literature retrieval process in this systematic review was described using the PRISMA 2020 flow chart (Fig. 1). The PRISMA_checklist can be found in **Supplementary Material 2**. A total of 517 relevant articles were identified from 12 databases; and after removing duplicates, 267 articles remained. Following preliminary screening of titles and abstracts, 230 articles that did not meet the inclusion criteria were excluded, leaving a final set of 37 articles. After full-text assessment, an additional 17 studies were excluded due to mismatched experiments or intervention methods, inconsistent outcome indicators, duplication or incomplete data. Ultimately, a total of 20 original studies met the inclusion criteria.

### 3.2 Characteristics of Included Studies

This study encompassed a total of 20 RCTs [17, 18, 19, 20, 21, 22, 23, 24, 25, 26, 27, 28, 29, 30, 31, 32, 33, 34, 35, 36], involving 2095 participants, out of which 984 individuals underwent Tai Chi intervention. The participants were of diverse gender and aged from 52 to 75 years old. The included papers were published from 2010 and 2022. Each of the twenty papers employed Tai Chi rehabilitation exercises as the primary intervention measure while the control group received either conventional treatment or a combination of conventional non-Tai Chi rehabilitation exercises (Table 1, Ref. [17, 18, 19, 20, 21, 22, 23, 24, 25, 26, 27, 28, 29, 30, 31, 32, 33, 34, 35, 36]).

**Table 1.** **Characteristics of included studies**.

Studies	Type of coronary disease	Tai Chi rehabilitation group			Control group			Outcomes
		Sample size (M/F)	Age	Intervention characteristics	Sample size (M/F)	Age	Control characteristics	
Sato *et al*. (2010) [17]	CHD	10 (6/4)	68.0 ± 5.0	24 Style Yang’s Tai Chi, 60 min per day for 48 weeks	10 (7/3)	68.0 ± 4.0	Blank	${\mathrm{VO}}_{2}$ max
Zhang and Chen X (2011) [18]	CHD	66 (40/26)	56.4 ± 4.3	42 Style Chen’s Tai Chi, more than 30 min, 5 times per week for 24 weeks	66 (42/24)	55.7 ± 4.2	Blank	6MWT
Huang (2014) [19]	CHD combined with heart failure	44 (21/23)	64.3 ± 6.5	Tai Chi ball-holding cloud hand, more than 30 min 5 times per week for 4 weeks	44 (23/21)	61.6 ± 7.1	Blank	6MWT
								NYHA class
Nery *et al*. (2015) [20]	AMI recovery period	31 (25/6)	56.0 ± 9.0	Wu’s Tai Chi, 60 min 3 times per week for 12 weeks	30 (19/11)	60.0 ± 9.0	Blank	${\mathrm{VO}}_{2}$ max
Sang *et al*. (2015) [21]	CHD combined with heart failure	50 (28/22)	65.3 ± 8.2	Tai Chi Rehabilitation Exercise, 15 min per day for 12 weeks	50 (29/21)	76.2 ± 7.5	Blank	6MWT
Wu *et al*. (2016) [22]	CHD	30 (21/9)	59.3 ± 6.9	24 Styles Simplified Tai Chi, 60 min 5 times per week for 12 weeks	30 (21/9)	58.9 ± 6.2	Blank	Anxiety Scale
								Depression Scale
Liu (2017) [23]	CHD combined with heart failure	33 (15/18)	55.2 ± 1.3	24 Styles Simplified Tai Chi, 60 min 3–4 times per week for 28 weeks	33 (16/17)	54.8 ± 1.3	Blank	6MWT
								NYHA class
Liu J *et al*. (2017) [24]	Post- PCI	40 (28/12)	55.2 ± 1.3	Cloud Hand Tai Chi, 50 min each time, 3–4 times a week for 12 weeks	40 (25/15)	54.8 ± 1.3	Walking, 50 min each time, 3–4 times a week for 12 weeks	6MWT
								PHC
								MHC
Deng *et al*. (2018) [25]	AMI combined with heart failure	57 (31/26)	64.7 ± 4.2	42 Style Chen’s Tai Chi, 30 min 5 times per week for 24 weeks	56 (29/27)	67.2 ± 4.9	Blank	6MWT
								Anxiety Scale
								Depression Scale
Jiang (2018) [26]	Chronic Stable Angina	61 (36/25)	54.5 ± 8.7	24 Style Tai Chi, 60 min 5 times per week for 12 weeks	65 (40/25)	55.4 ± 10.4	Walking or cycling, 60 minutes 3 times per week for 12 weeks	${\mathrm{VO}}_{2}$ max
								Anxiety Scale
								Depression Scale
Liu (2018) [27]	Chronic Stable Angina	46 (24/22)	68.6 ± 5.2	24 Styles Simplified Tai Chi, 90 min 3 times per week for 12 weeks	44 (26/18)	69.7 ± 5.5	Walking, 60 minutes 3 times per week for 12 weeks	6MWT
Li *et al*. (2019) [28]	CHD	163 (72/91)	63.6 ± 6.6	24 Style Yang’s Tai Chi, 60 min per day for 48 weeks	163 (68/95)	65.4 ± 5.7	Blank	Anxiety Scale
								Depression Scale
								PHC
								MHC
Liu *et al*. (2020) [29]	Post- PCI	30 (24/6)	60.4 ± 10.9	24 Style Tai Chi, 50–60 min each time, twice a day, 5 times a week for 12 weeks	31 (25/5)	57.0 ± 11.0	Blank	PHC
								MHC
Ma *et al*. (2020) [30]	CHD	15 (10/5)	61.4 ± 11.7	24 Style Yang’s Tai Chi, 60 min 3 times per week for 12 weeks	15 (11/4)	67.3 ± 8.4	Blank	6MWT
								NYHA class
								PHC
								MHC
Song and YI (2020) [31]	Post- PCI	70 (39/31)	65.4 ± 8.8	24 Styles Simplified Tai Chi, 30 min 3 times per week for 24 weeks	72 (43/29)	64.2 ± 9.0	Blank	6MWT
								PHC
								MHC
Yu *et al*. (2020) [32]	AMI combined with heart failure	100 (52/48)	67.7 ± 5.8	24 Styles Simplified Tai Chi, 20 min each time, twice a day for 24 weeks	100 (54/46)	68.3 ± 6.4	Blank	6MWT
								NYHA class
Yu *et al*. (2021) [33]	Post- PCI	28 (14/14)	65.4 ± 4.1	Unknown Tai Chi types and exercise prescriptions, Duration 1 year	30 (17/13)	66.3 ± 5.4	Blank	6MWT
Yang (2021) [34]	AMI recovery period	69 (36/33)	63.2 ± 5.6	Tai Chi Ball, 30 min per day for 4 weeks	69 (35/34)	64.1 ± 5.6	Blank	${\mathrm{VO}}_{2}$ max
Zhang *et al*. (2022) [35]	CHD	23 (19/4)	56.9 ± 10.5	24 Styles Simplified Tai Chi, 60 min 6 times per week for 36 weeks	24 (17/7)	59.0 ± 8.9	Blank	PHC
								MHC
Lyu *et al*. (2022) [36]	CHD	18 (14/4)	56.7 ± 9.4	24 Styles Simplified Tai Chi, 60 min 3 times per week for 44 weeks	12 (10/2)	57.4 ± 10.4	Warm-up exercises, aerobic exercises, resistance exercises, and relaxation, 60 min 3 times per week for 44 weeks	Anxiety Scale
								Depression Scale

Abbreviations: AMI, acute myocardial infarction; CHD, coronary heart disease; PCI, percutaneous coronary intervention; 6MWT, six-minute walk test; NYHA class, New York Heart Association; ${\mathrm{VO}}_{2}$ max, maximal oxygen consumption; PHC, physiological health components; MHC, mental health components; M/F, male/female.

### 3.3 Risk of Bias Assessment

**Fig. 2.**
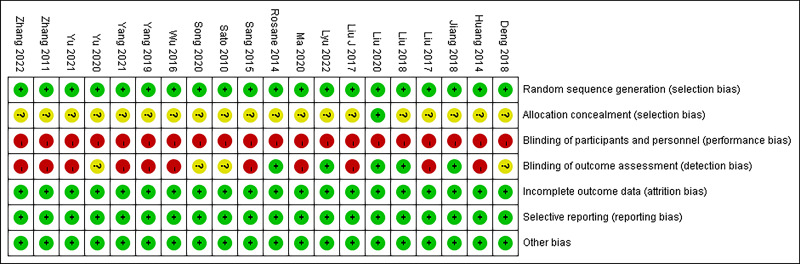
**Risk of bias: study quality was assessed according to the revised Cochrane risk of bias tool for randomized trials**.

**Fig. 3.**
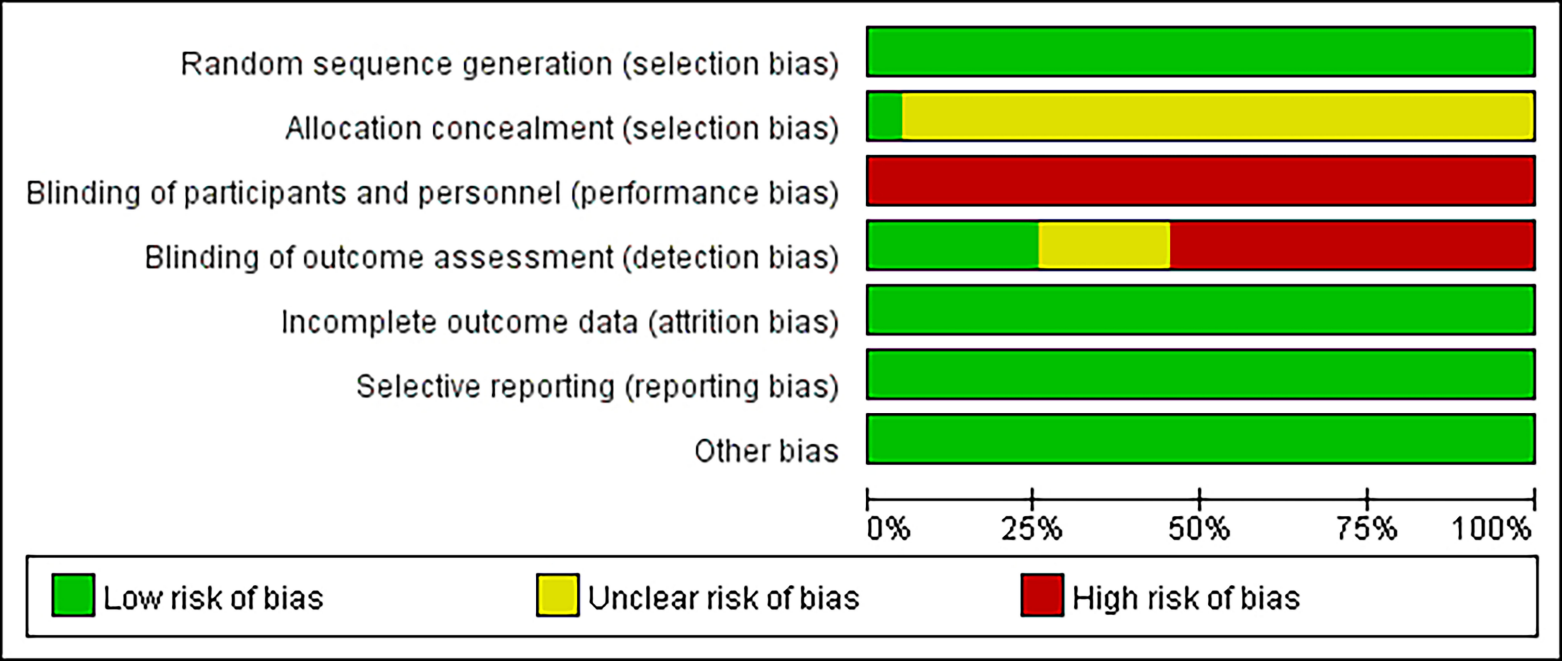
**Risk of bias: study quality was assessed according to the revised Cochrane risk of bias tool for randomized trials**.

The Cochrane risk of bias assessment tool was employed to individually evaluate each literature for the following aspects: methods of random assignment, concealment of allocation scheme, blinding of patients and implementers, blinding of outcome reviewers, completeness of outcome data, selective reporting of study results, and other sources of bias (Fig. 2, Fig. 3). Initially, all 20 literatures exhibited comparability (*p* > 0.05). Among these studies, fifteen specified the method of random assignment with 12 utilizing a random number table, 2 employing random sampling, and 1 adopting a specific type of draw. Conversely, the remaining five studies did not provide explicit details regarding their methodological approach to random assignment. Only one study reported on the allocation concealment in trials while another study implemented a single-blinded experimental design. All studies maintained complete outcome data without any instances of selective reporting; however none out of the twenty studies provided an accompanying description to assess potential presence or absence of other biases.

### 3.4 Meta-Analysis for Physical Function Outcomes Measures

#### 3.4.1 6MWT

**Fig. 4.**
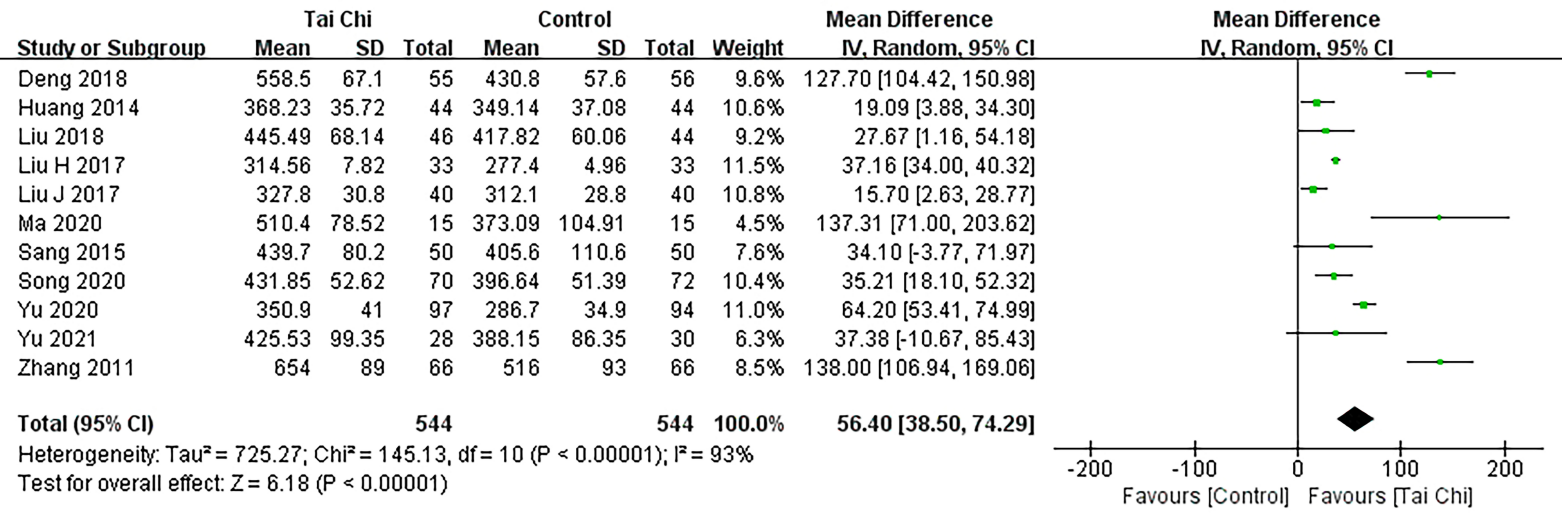
**Forest plot: 6MWT.** 6MWT, six-minute walk test; SD, standard difference; 95% CI, 95% confidence interval; Df, degree of freedom; IV, inverse variance.

The study examined 11 literature sources [18, 19, 21, 23, 24, 25, 27, 30, 31, 32, 33], encompassing a total of 171 participants, which reported the outcome of the 6MWT. A meta-analysis using random effects pooling revealed (Fig. 4) that the Tai Chi intervention group exhibited a significant improvement in the distance covered during the 6MWT among CHD patients compared to the control group (MD 56.40; 95% CI: 38.50–74.29, *p* < 0.001).

#### 3.4.2 ${\mathrm{VO}}_{2}$ Max

**Fig. 5.**
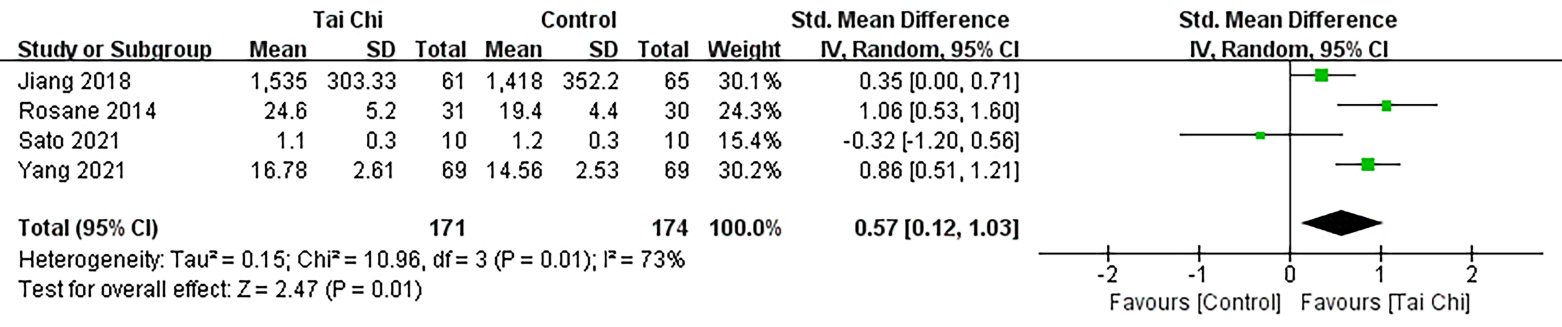
**Forest plot: ${\mathrm{VO}}_{2}$ max.** SD, standard difference; 95% CI, 95% confidence interval; Df, degree of freedom; Std, standard deviation; IV, inverse variance.

The study conducted a meta-analysis of four literature sources [17, 20, 26, 34], which included a total of 171 subjects, that reported the outcome of ${\mathrm{VO}}_{2}$ max. The results from a random effects pooled meta-analysis indicated that (Fig. 5) the Tai Chi group exhibited a significant improvement in ${\mathrm{VO}}_{2}$ max compared to the control group among CHD patients (SMD = 0.57; 95% CI: 0.12–1.03, *p* = 0.01).

#### 3.4.3 NYHA Class

**Fig. 6.**
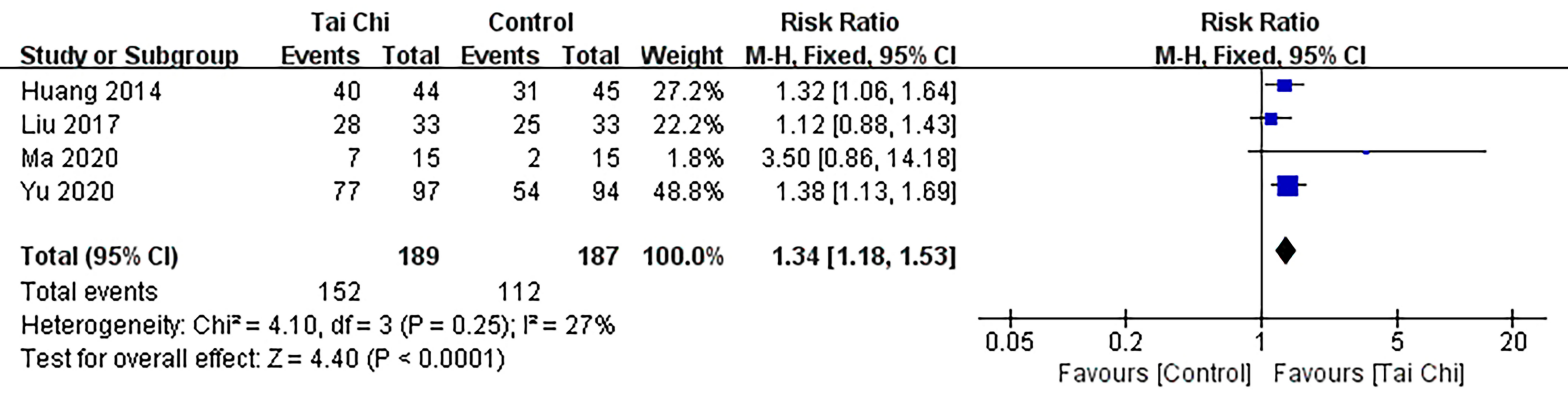
**Forest plot: NYHA class.** 95% CI, 95% confidence interval; Df, degree of freedom; NYHA, New York heart association; M-H, mantel-haenszel.

The meta-analysis included a total of 376 subjects from four studies [19, 23, 30, 32] examining the impact of Tai Chi on NYHA class levels. Our findings, presented in Fig. 6 using fixed effects pooling, revealed a significant increase in NYHA class levels among patients in the Tai Chi group compared to the control group (RR 1.34; 95% CI: 1.18–1.53, *p* < 0.001).

#### 3.4.4 PHC

**Fig. 7.**
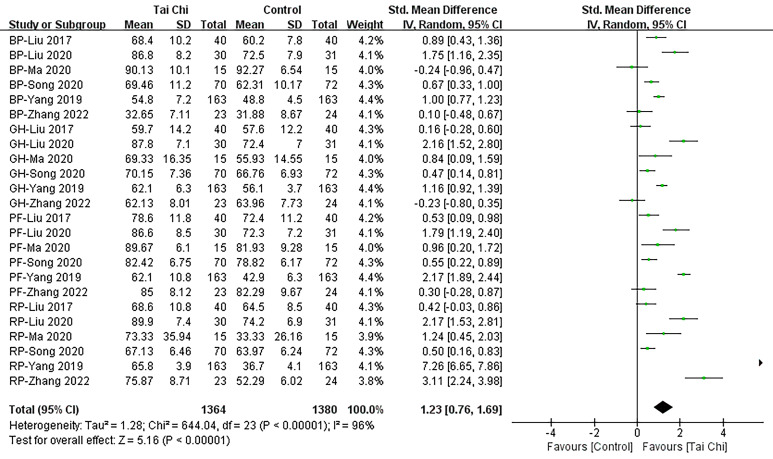
**Forest plot: PHC.** SD, standardized difference; 95% CI, 95% confidence interval; Df, degree of freedom; Std, standard deviation; PHC, physical health components; IV, inverse variance.

A total of 686 subjects were included in the analysis, encompassing 6 studies [24, 28, 29, 30, 31, 35] investigating the impact of Tai Chi on PHC levels. Utilizing a random effects pooled meta-analysis (Fig. 7) our findings revealed a significant increase in PHC levels within the Tai Chi group compared to the control group (SMD = 1.23; 95% CI: 0.76–1.69, *p* < 0.001).

#### 3.4.5 Physical Function Evidence Level Evaluation

The Gradepro software (version 3.6, Cochrane Groups, Cavendish, London, UK) of provides evidence ratings for 6MWT, ${\mathrm{VO}}_{2}$ max, NYHA class, and PHC scores. Among these variables, the evidence ratings for 6MWT, NYHA class, and PHC were categorized as intermediate while the evidence rating for ${\mathrm{VO}}_{2}$ max was classified as low. Tai Chi rehabilitation exercise demonstrates significant efficacy in enhancing patient physiology (Table 2).

**Table 2.** **Physical function evidence level evaluation**.

Quality assessment							No of patients		Effect		Quality	Importance
No of studies	design	Risk of bias	inconsistency	Indirectness	Imprecision	Other considerations	Tai Chi rehabilitation exercise	Control	Relative (95% CI)	Absolute		
Six-minutes walking test (Better indicated by lower values)												
6	randomised trials	${\mathrm{serious}}^{1}$	no serious inconsistency	no serious indirectness	no serious imprecision	none	278	280	-	MD 23.16 higher (15.29 to 31.04 higher)	⊕⊕⊕ MODERATE	CRITICAL
Peak Oxygen Uptake (Better indicated by lower values)												
4	randomised trials	${\mathrm{serious}}^{1}$	${\mathrm{serious}}^{2}$	no serious indirectness	no serious imprecision	none	171	174	-	SMD 0.57 higher (0.12 to 1.03 higher)	⊕⊕ LOW	CRITICAL
Heart function grade of NYHA												
4	randomised trials	${\mathrm{serious}}^{1}$	no serious inconsistency	no serious indirectness	no serious imprecision	none	152/189 (80.4%)	112/187 (59.9%)	RR 1.34 (1.18 to 1.53)	204 more per 1000 (from 108 more to 317 more)	⊕⊕⊕ MODERATE	IMPORTANT
								63.2%		215 more per 1000 (from 114 more to 335 more)		
Physical health components score (Better indicated by lower values)												
6	randomised trials	${\mathrm{serious}}^{1}$	${\mathrm{serious}}^{3}$	no serious indirectness	no serious imprecision	strong ${\mathrm{association}}^{4}$	341	345	-	SMD 1.23 higher (0.76 to 1.69 higher)	⊕⊕⊕ MODERATE	IMPORTANT

^1^Tai Chi cannot be blinded as an intervention, and the allocation scheme conceals uncertainty. ^2^Peak oxygen uptake was measured in different units. ^3^The evaluation scales are not the same. ^4^The effect size d = 1.23 reached a large effect. The more ⊕ symbols, the higher the quality of the literature. 95% CI, 95% confidence interval; MD, mean difference; SMD, standardized mean difference; NYHA, New York heart association.

### 3.5 Meta-Analysis for Psychological Health Outcomes Measures

#### 3.5.1 Anxiety Scales

**Fig. 8.**
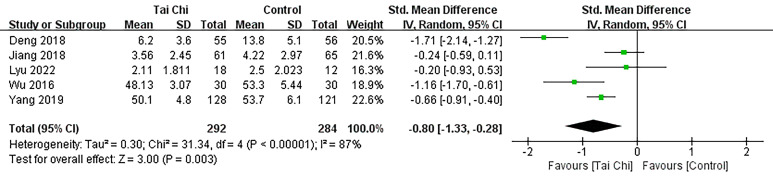
**Forest plot: Anxiety Scales.** SD, standardized difference; 95% CI, 95% confidence interval; Df, degree of freedom; Std, standard deviation; IV, inverse variance.

A total of 576 subjects were included in the analysis from five literature sources [22, 25, 26, 28, 36] investigating the impact of Tai Chi on anxiety levels measured by Anxiety Scales in CHD patients. The results (Fig. 8) demonstrated a significant reduction in anxiety levels among CHD patients who underwent Tai Chi compared to those in the control group (standardized mean difference (SMD) –0.80; 95% CI: –1.33–0.28, *p* = 0.003).

#### 3.5.2 Depression Scales

**Fig. 9.**
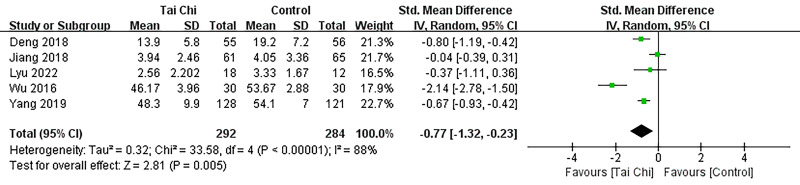
**Forest plot: Depression Scales.** SD, standardized difference; 95% CI, 95% confidence interval; Df, degree of freedom; Std, standard deviation; IV, inverse variance.

A total of 576 subjects were included in the meta-analysis, which incorporated findings from five literature sources [22, 25, 26, 28, 36] assessing Depression Scales. The results (Fig. 9) revealed that the Tai Chi intervention exhibited significant efficacy compared to the control group for alleviating depression among patients with coronary heart disease. (SMD –0.77; 95% CI: –1.32–0.23, *p* = 0.005).

#### 3.5.3 MHC

**Fig. 10.**
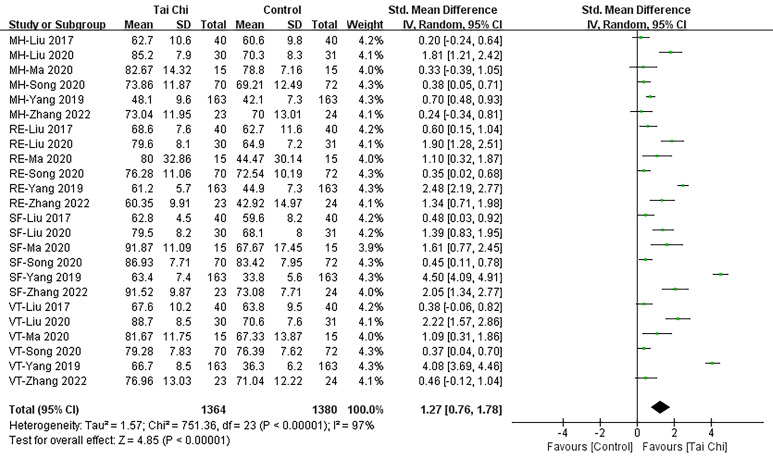
**Forest plot: MHC.** SD, standardized difference; 95% CI, 95% confidence interval; Df, degree of freedom; Std, standard deviation; MHC, mental health components; IV, inverse variance.

A total of 686 subjects were included in the analysis, encompassing 6 studies [24, 28, 29, 30, 31, 35] investigating the impact of Tai Chi on mental health outcomes. A random-effects meta-analysis revealed (Fig. 10) a significant improvement in mental health among participants in the Tai Chi group compared to those in the control group (SMD = 1.27; 95% CI: 0.76–1.78, *p* < 0.001).

#### 3.5.4 Psychological Health Evidence Level Evaluation

Gradepro provides evidence-based rating scales for anxiety, depression, and mental health. The level of evidence for the anxiety scale is low, while the scores on the depression scale are extremely low. The grade of evidence for mental health falls in the intermediate range. Tai Chi rehabilitation exercise is highly likely to improve patient mental health (Table 3).

**Table 3.** **Psychological health evidence level evaluation**.

Quality assessment							No of patients		Effect		Quality	Importance
No of studies	design	Risk of bias	inconsistency	Indirectness	Imprecision	Other considerations	Tai Chi rehabilitation exercise	Control	Relative (95% CI)	Absolute		
Anxiety (Better indicated by lower values)												
5	randomised trials	${\mathrm{serious}}^{1}$	very ${\mathrm{serious}}^{2}$	no serious indirectness	no serious imprecision	strong ${\mathrm{association}}^{3}$	292	284	-	SMD 0.8 lower (1.33 to 0.28 lower)	⊕⊕ LOW	CRITICAL
Depression (Better indicated by lower values)												
5	randomised trials	${\mathrm{serious}}^{1}$	very ${\mathrm{serious}}^{2}$	no serious indirectness	no serious imprecision	none	292	284	-	SMD 0.77 lower (1.32 to 0.23 lower)	⊕ VERY LOW	CRITICAL
Mental Health Components Score (Better indicated by lower values)												
6	randomised trials	${\mathrm{serious}}^{1}$	${\mathrm{serious}}^{2}$	no serious indirectness	no serious imprecision	strong ${\mathrm{association}}^{4}$	341	345	-	SMD 1.27 higher (0.76 to 1.78 higher)	⊕⊕ MODERATE	IMPORTANT

^1^Tai Chi cannot be blinded as an intervention, and the allocation scheme conceals uncertainty. ^2^Evaluation scales are not the same. ^3^The effect amount d = 0.8 reached the large effect. ^4^The effect size d = 1.27 reached the large effect. The more ⊕ symbols, the higher the quality of the literature. 95% CI, 95% confidence interval; SMD, standardized mean difference.

## 4. Discussion

Currently, cardiovascular diseases are the primary health concern, with coronary heart disease being the leading cause of motality [1]. Preventing cardiovascular diseases and improving patients’ quality of life has become a significant public health priority. The Tai Chi Cardiac Rehabilitation Programme is a Chinese exercise regimen that offers flexibility in time and location, making it an ideal choice for patients with coronary heart disease to practice, particularly suitable for home-based exercise [7]. Compared with conventional exercise rehabilitation programmes, the Tai Chi Heart Health Programme is more culturally appropriate, cost-effective, feasible, and highly adherent [37]. Therefore, integrating Tai Chi with modern cardiac rehabilitation concepts holds immense practical significance and theoretical value. This integration allows us to fully harness the advantages of traditional Chinese sports and health maintenance techniques while constructing a safe and effective Taijiquan cardiac rehabilitation programme. We conducted a systematic evaluation of the effects of tai chi on the physical function and mental well-being among patients. Meta-analyses results indicate that tai chi is more effective than conventional treatment in improving both physical and mental health outcomes.

We analyzed the outcome indicators for patients with coronary artery disease including 6MWT, ${\mathrm{VO}}_{2}$ Max, NYHA class, and PHC scores. Our findings suggest that Tai Chi exercise can enhance aerobic work capacity in patients, ameliorate myocardial ischaemia symptoms and ischemic events, ultimately leading to improve cardiac function and activity tolerance levels. Additionally, patient’s quality of life of is closely associated with their cardiac function status. Tai Chi’s gentle movements and steady rhythm promote relaxation and improve blood supply to the heart, compared with traditional exercise workouts [7]. Rhythmic respiratory control also improves hypoxia, reducing symptoms and achieving a better quality of life. Wang *et al*. [38] and Chen [37] also utilized 6MWT and ${\mathrm{VO}}_{2}$ max as outcome measures; However, due to the limited available literature for descriptive analysis, their results were consistent with our study’s finding-Tai Chi effectively improves exercise tolerance. While NYHA class primarily assesses cardiac function in CHD patients with heart failure, PHC scores provide a more comprehensive evaluation across four dimensions: PF, RP, BP, and GH. To date, no related systematic review has analyzed NYHA class and PHC scores. Our study demonstrates that Tai Chi intervention significantly improves cardiac function in CHD patients while effectively enhancing PHC scores.

Additionally, the present study shows that Tai Chi exercise reduces anxiety levels and depression symptoms while increasing MHC scores among CHD patients. Similarly Liu *et al*. [39] assessed the effects of Tai Chi on anxiety and depression in patients with such conditions. The results showed that the Tai Chi group had significantly lower scores for anxiety (SMD 9.28, 95% CI: 17.46–1.10, *p* = 0.03) and depression (SMD 9.42, 95% CI: 13.59–5.26, *p* < 0.001) compared to the non-exercise control group. However, it should be noted that this meta-analysis included studies that was not limited to randomized controlled experiments and the number of studies was small. The practice of Tai Chi requires psychological focus concentration on guiding actions while maintaining a relaxed state of mind, which can induce protective inhibition in certain areas of the cerebral cortex allowing them to rest effectively [38, 40]. Additionally, Tai Chi exercises can activate emotions and regulate brain function by promoting long-term persistence in practice leading to recovery and improvement in cerebral function as well as alleviating various chronic diseases caused by nervous system disorders.

Furthermore, according to Traditional Chinese medicine theory, Tai Chi is a form of movement that combines both active phases with periods of rest within each movement sequence resulting in virtual and actual transformation occurring simultaneously within the body cavity. A person’s abdominal muscles become loose and elastic during movement, and blood circulation in the abdominal bore is unobstructed during movement, thereby improving the metabolism of the visceral organs and facilitating the patency of the arteries and veins in the body cavity [7]. From a modern medical perspective supported by pathophysiological evidence, it has been demonstrated that Tai Chi exercise enhances endothelial function by increasing endothelium-dependent vasodilation while reducing arterial stiffness among patients, moreover it also reduces expression levers of inflammatory mediators [41]. All these theoretical foundations provide strong support for the safety profile associated with Tai Chi rehabilitation exercises since no significant complications were reported from any included studies while compliance rate remained satisfactory. Therefore, we recommend Tai Chi exercises as an adjunctive therapy for cardiac rehabilitation in patients with coronary heart disease. However, definitive conclusions cannot be drawn yet at present due to the presence of inter-study heterogeneity and limited sample sizes. Consequent, further large-sample randomized controlled trials with rigorously designs are warranted to substantiate any conclusive findings. This systematic review encompasses physical activity intervention studies subsequent to cardiac rehabilitation.

### Study Limitations

There are several limitations to the present study. Firstly, despite conducting a more comprehensive search across 12 databases, the total sample size of these 20 studies remained small. A smaller sample size in experimental analysis may potentially amplify the effects of Tai Chi intervention compared to larger sample analysis. Secondly, Our RCTs also exhibited methodological differences in random allocation, concealment of allocation schemes, and blinding procedures. Due to the specificity of interventions, individual studies did not clearly elucidate the allocation method; most studies did not mention the allocation concealment scheme which could lead to overestimate of the treatment effects and hinder achieving double-blinding conditions. Thirdly, there is no standardized approach for the Tai Chi interventions within this meta-analysis; variations exist in design aspects such as type, duration, frequency of exercise sessions, intervention period length, prior experience of Tai Chi coaches and participants’ mastery levers. These discrepancies may contribute to potential sources of heterogeneity. Lastly, due to limited inclusion literature for each outcome measure and absence of a funnel plot test for publication bias assessment purposes; we conducted grade grading based on articles with very low to moderate grades of evidence while anticipating higher grades for updated findings.

## 5. Conclusions and Outlook

Despite the limitations inherent in this study, the findings hold significant implications for clinical practice. Tai Chi rehabilitation exercise has demonstrated its potential to enhance both physical function and mental well-being among patients with coronary artery disease, thereby serving as a promising adjunctive therapy for cardiac rehabilitation during later stages of life. Future investigations should aim to compare CHD patients across different levers of cardiac function and develop standardized intervention protocols to provide tailored exercise recommendations for individual patients. Furthermore, it is highly recommended that a globally standardized and unified database be established to ensure quality control in Tai Chi research, ultimately elevating the level of evidence from Tai Chi trials and further substantiating its value in disease prevention and treatment.

## Abbreviations

CVD, cardiovascular disease; CHD, coronary heart disease; RCTs, randomized controlled trials; 6MWT, six-minute walk test; ${\mathrm{VO}}_{2}$ max, maximal oxygen consumption; NYHA, New York Heart Association; PHC, physical health components; MHC, Mental Health Components; MACEs, major adverse cardiovascular events; PF, physical function; RP, role physical; BP, bodily pain; GH, general health; VT, vitality; SF, social function; RE, emotional function; MH, mental health; ROB, risk of bias; TCM, traditional chinese medicine.

## Supplementary Material

Supplementary material associated with this article can be found, in the online version, at https://doi.org/10.31083/j.rcm2506227.
