# Supplementary material for: Effect of Tai Chi-Based Psychosomatic Rehabilitation Exercise on Physiological Function and Mental Health of Patients with Coronary Heart Disease: A Meta-Analysis
Source: Rev Cardiovasc Med. 2024 Jun 24;25(6):227. doi: 10.31083/j.rcm2506227 (PMC11270087; doi:10.31083/j.rcm2506227)
Supplement: Supplementary file 1 [file 2153-8174-25-6-227-s1.zip › Supplementary Material 1.pdf]

Appendix A of Supplementary Materials

#1 ("Coronary Disease"[Mesh]) OR ((((((((Coronary Diseases[Title/Abstract]) OR (Disease, Coronary[Title/Abstract])) OR (Diseases, Coronary[Title/Abstract])) OR (Coronary Heart Disease[Title/Abstract])) OR (Coronary Heart Diseases[Title/Abstract])) OR (Disease, Coronary Heart[Title/Abstract])) OR (Diseases, Coronary Heart[Title/Abstract])) OR (Heart Disease, Coronary[Title/Abstract])) OR (Heart Diseases, Coronary[Title/Abstract]))

#2 ("Coronary Disease"[Mesh]) OR ((((((((Coronary Diseases[Title/Abstract]) OR (Disease, Coronary[Title/Abstract])) OR (Diseases, Coronary[Title/Abstract])) OR (Coronary Heart Disease[Title/Abstract])) OR (Coronary Heart Diseases[Title/Abstract])) OR (Disease, Coronary Heart[Title/Abstract])) OR (Diseases, Coronary Heart[Title/Abstract])) OR (Heart Disease, Coronary[Title/Abstract])) OR (Heart Diseases, Coronary[Title/Abstract]))

#3 ("Percutaneous Coronary Intervention"[Mesh]) OR (((((((((((Coronary Intervention, Percutaneous[Title/Abstract]) OR (Coronary Interventions, Percutaneous[Title/Abstract])) OR (Intervention, Percutaneous Coronary[Title/Abstract])) OR (Interventions, Percutaneous Coronary[Title/Abstract])) OR (Percutaneous Coronary Interventions[Title/Abstract])) OR (Percutaneous Coronary Revascularization[Title/Abstract])) OR (Coronary Revascularization, Percutaneous[Title/Abstract])) OR (Coronary Revascularizations, Percutaneous[Title/Abstract])) OR (Percutaneous Coronary Revascularizations[Title/Abstract])) OR (Revascularization, Percutaneous Coronary[Title/Abstract])) OR (Revascularizations, Percutaneous Coronary[Title/Abstract]))

#4 ("Coronary Artery Disease"[Mesh]) OR ((((((((((((((Artery Disease, Coronary[Title/Abstract]) OR (Artery Diseases, Coronary[Title/Abstract])) OR (Coronary Artery Diseases[Title/Abstract])) OR (Left Main Coronary Artery Disease[Title/Abstract])) OR (Left Main Disease[Title/Abstract])) OR (Left Main Diseases[Title/Abstract])) OR (Left Main Coronary Disease[Title/Abstract])) OR (Coronary Arteriosclerosis[Title/Abstract])) OR (Arterioscleroses, Coronary[Title/Abstract])) OR (Coronary Arterioscleroses[Title/Abstract])) OR (Atherosclerosis, Coronary[Title/Abstract])) OR (Atheroscleroses, Coronary[Title/Abstract])) OR (Coronary Atheroscleroses[Title/Abstract])) OR (Coronary Atherosclerosis[Title/Abstract])) OR (Arteriosclerosis, Coronary[Title/Abstract]))

#5 ("Myocardial Infarction"[Mesh]) OR ((((((((((Infarction, Myocardial[Title/Abstract]) OR (Infarctions, Myocardial[Title/Abstract])) OR (Myocardial Infarctions[Title/Abstract])) OR (Cardiovascular Stroke[Title/Abstract])) OR (Cardiovascular Strokes[Title/Abstract])) OR (Stroke, Cardiovascular[Title/Abstract])) OR (Strokes, Cardiovascular[Title/Abstract])) OR (Myocardial Infarct[Title/Abstract])) OR (Infarct, Myocardial[Title/Abstract])) OR (Infarcts, Myocardial[Title/Abstract])) OR (Myocardial Infarcts[Title/Abstract]))

#6 ("Coronary Artery Bypass"[Mesh]) OR ((((((((((((Artery Bypass, Coronary[Title/Abstract]) OR (Artery Bypasses, Coronary[Title/Abstract])) OR (Bypasses, Coronary Artery[Title/Abstract])) OR (Coronary Artery Bypasses[Title/Abstract])) OR (Coronary Artery Bypass Surgery[Title/Abstract])) OR (Bypass, Coronary Artery[Title/Abstract])) OR (Coronary Artery Bypass Grafting[Title/Abstract])) OR (Aortocoronary Bypass[Title/Abstract])) OR (Aortocoronary Bypasses[Title/Abstract])) OR (Bypass, Aortocoronary[Title/Abstract])) OR (Bypasses, Aortocoronary[Title/Abstract])) OR (Bypass Surgery, Coronary Artery[Title/Abstract]))

#7 ("Tai Ji"[Mesh]) OR (((((((Taiji[Title/Abstract]) OR (Tai-ji[Title/Abstract])) OR (Tai ji[Title/Abstract])) OR (Taichi[Title/Abstract])) OR (Tai Chi[Title/Abstract])) OR (Tai chi quan[Title/Abstract])) OR (Tai chi chuan[Title/Abstract]))

#8 randomized controlled trial[Publication Type] OR randomized[Title/Abstract] OR placebo[Title/Abstract]

#9 #1 OR #2 OR #3 OR #4 OR #5 OR #6 OR #7

#10 #7 AND #8 AND #9
